# Stem Cells in Cosmetic Applications: Advancements, Challenges, and Future Directions

**DOI:** 10.1111/jocd.70866

**Published:** 2026-05-05

**Authors:** Farid KarkonShayan, Nasim Saderi, Iman Morshedi, Behnaz Ghamari, Sepideh KarkonShayan

**Affiliations:** ^1^ Tabriz University of Medical Science Tabriz Iran; ^2^ Faculty of Pharmacy Islamic Azad University, Tehran Medical Branch Tehran Iran; ^3^ İstanbul Tıp fakültesi University İstanbul Turkey; ^4^ Clinical Research Development Unit of Tabriz Valiasr Hospital Tabriz University of Medical Sciences Tabriz Iran

**Keywords:** biological products, cosmetics, plant stem cells, skin regeneration, ultraviolet protection

## Abstract

**Background:**

Biotechnology has significantly impacted the cosmetics industry, particularly through the incorporation of plant stem cells, which possess regenerative properties beneficial for skincare.

**Aims:**

This review aims to evaluate the potential benefits and challenges of using plant stem cells in cosmetics, while discussing future directions for their application in skin care products.

**Patients/Methods:**

The review synthesizes existing literature on the regenerative capabilities of plant stem cells, their role in combating aging, promoting skin repair, and providing protection against ultraviolet damage. It also examines biotechnological methods such as plant cell cultures that mitigate issues associated with raw plant materials.

**Results:**

Plant stem cells demonstrate self‐renewal and differentiation capabilities, crucial for tissue regeneration and skin healing. Despite their advantages, the cosmetic industry faces hurdles including inconsistent terminology, regulatory challenges, high production costs, and the prevalence of marketing strategies lacking clinical validation.

**Conclusions:**

While plant stem cell technology offers promising advancements in skincare, the cosmetic industry must address regulatory and validation concerns to ensure consumer safety and product efficacy. Future research should focus on establishing standardized practices and clinical trials to substantiate the claims of stem cell‐based products in cosmetics.

**Abbreviations**
DNA: deoxyribonucleic acid
IL‐6: interleukin 6
LACCE: *Leontopodium alpinum* callus culture extract
LPS: lipopolysaccharide
LSCECC: leaf stem cell extract of *Coffea canephora*

RSCEs: *Rosa damascena* stem cell–derived exosomes
RSCs: *Rosa damascena* stem cells
UV: ultraviolet
UV‐A: ultraviolet A
UV‐B: ultraviolet B
VEGF: vascular endothelial growth factor

## 1 Introduction

Biotechnology has greatly affected the cosmetic field by creating new ways to produce active ingredients that can slow down skin aging [1]. One important research area today is plant stem cells, especially their use in cosmetic product formulation and their effects on the skin [2]. Plants have a powerful regenerative ability, allowing them to repair damaged tissues and even grow into new plants under stressful conditions [3]. In 1902, the Austrian botanist Gottlieb Haberlandt first explained that callus tissue can form from fully developed plant cells and suggested that every plant cell can regenerate a whole plant. Later research showed that auxins help in forming roots from callus, while cytokinins are mainly responsible for stem formation [4]. Plant stem cells are located in specific regions called meristems, which are divided into primary and secondary meristems. The primary meristems include apical, intercalary, and germ meristems, while secondary meristems include cambium, phellogen, and callus [5]. One of the main control systems for plant stem cell growth works through the interaction between WUSCHEL and CLAVATA3 proteins in the apical meristem. The WUS protein has an important role in converting normal plant cells back into stem cells [6].

Stem cells are undifferentiated cells characterized by self‐renewal and multi‐lineage differentiation, properties that underpin their central role in regenerative medicine [7]. They can renew themselves and differentiate into specialized cell types, which is important for maintaining tissue health in many organisms [8]. Their regenerative ability helps repair damaged cells and supports tissue balance in the body [9]. These biological features have attracted significant interest in aesthetic and cosmetic applications, where the restoration and regeneration of tissue structure and function are the main goals. In regenerative dermatology and cosmetic surgery, stem cell‐based approaches aim to address structural and functional deterioration associated with aging, injury, or cosmetic alteration, promoting cellular mechanisms that contribute to tissue repair, neovascularization, and extracellular matrix synthesis [10].

Plant stem cells have been linked to many positive effects, such as increasing the activity and lifespan of fibroblasts like in *Gardenia jasminoides* and *Oryza sativa* [11], improving skin flexibility using plants such as *Capsicum annuum* , *Symphytum officinale* , and *Opuntia* species [12], controlling cell division and aiding with the repair of damaged skin cells like with *Opuntia* species and *Panax ginseng* [13], supporting DNA repair and reducing oxidative stress using plants such as *Lycopersicon esculentum* , *Rubus ideaus*, and *Citrus limon* , and also protecting the skin from UV damage using *Opuntia ficus indica* and *Dolichos biflorus* [14].

Among the various stem cell types studied, mesenchymal stem cells (MSCs) have emerged as the most extensively investigated for cutaneous and aesthetic indications due to their relative ease of harvest, multipotency, and immunomodulatory properties. MSCs show their therapeutic effects through both direct differentiation into skin‐related lineages and paracrine signaling, whereby secreted growth factors and cytokines stimulate endogenous repair pathways, modulate inflammation, and enhance matrix formation (e.g., collagen and elastin) critical for skin rejuvenation and wound healing [15, 16]. In addition to direct cell‐based therapies, the concept of stem cell‐derived secretomes is gaining prominence, offering potential cosmetic benefits without the regulatory and safety complexities associated with administering living cells [17].

The use of whole plants in cosmetics has faced many challenges, including slow growth, dependence on seasonal harvesting, unstable levels of active compounds, and the presence of toxic metabolites, which limit their wider application [18]. Plant cell culture methods provide a good solution to these problems by allowing plant cells, tissues, or organs to grow under controlled, sterile, and nutrient‐rich conditions [18]. This technique enables the production of biologically active compounds that are rare in nature or difficult to synthesize chemically. As a result, plant stem cell extracts produced by this method are now widely used in both daily and professional cosmetic products. Examples include safflower pigments and saflorin from *Carthamus tinctorius* , and whitening agents such as arbutin from *Catharanthus roseus* or rose periwinkle [19]. The production process starts with choosing the proper plant material, followed by sterilization, callus induction, and repeated subculturing in a suitable medium such as Murashige and Skoog [20]. The cell line that shows the highest biomass production and fastest growth is selected for large‐scale production [21]. High‐pressure homogenization is then used to break the cells and release the active compounds. After that, the plant stem cell extracts are encapsulated in different carrier systems to improve their delivery in cosmetic products [22].

Despite promising preclinical results and early clinical reports, translating stem cell technologies into routine cosmetic practice faces several challenges, including a substantial gap in high‐quality clinical evidence, limitations in standardized protocols for cell processing and delivery, and regulatory uncertainty regarding safety and efficacy [23, 24]. Moreover, most marketed cosmetic products claim benefits related to “stem cells” without robust scientific validation of mechanism or outcome; a concern that underscores the need for caution and rigorous research [25]. This review critically synthesizes current scientific evidence on stem cell–based strategies for cosmetic applications, highlighting recent advancements, existing limitations, and emerging directions to inform safe and effective clinical translation.

## 2 Plant Stem Cell Technology in the Global Cosmetic Industry

Today, many pharmaceutical and cosmetic companies use bioreactors to produce innovative products that meet high‐quality standards. One of the most important techniques is in vitro cell culture, which is used to multiply plant stem cells for cosmetic purposes. The process usually begins by selecting a specific plant part, such as leaves, fruits, or roots. These parts are processed, and the extracted stem cells are placed on agar plates to induce callus formation [26]. When transferred into liquid culture, the cells continue to grow and multiply. For large‐scale production, specialized bioreactors are used to maintain stable, controlled growth conditions [27]. After sufficient growth, stem cells are subjected to high pressure to disrupt their membranes and release their bioactive compounds into the surrounding medium [28].

For example, Unhwa Corp. developed and patented an antiaging and antioxidant cosmetic composition derived from a ginseng cambium plant stem cell line. This product shows antioxidant effects by reducing the formation of reactive oxygen species, which are among the main causes of skin aging due to UV exposure. Studies also showed that leaf extracts from ginseng plants of different ages had varying antioxidant activity, which was correlated with higher levels of ginsenosides in older plants [29, 30]. The use of bioreactor cultures has also expanded to other plants such as *Haberlea rhodopensis, Rosa damascena, and Calendula officinalis* [14]. A well‐known example is the work of Mibelle Biochemistry, which introduced apple stem cell liposomes from the rare Swiss apple variety *Uttwiler Spätlauber*. This product, known as PhytoCellTec *Malus domestica* , showed clear anti‐wrinkle effects in clinical studies, reducing wrinkle depth by about 8% after 2 weeks and 15% after 4 weeks of use. Mibelle has also produced other plant stem cell extracts from *Argania spinosa*, *Saponaria pumila*, and *Vitis vinifera* for cosmetic applications [18].

**FIGURE 1:**
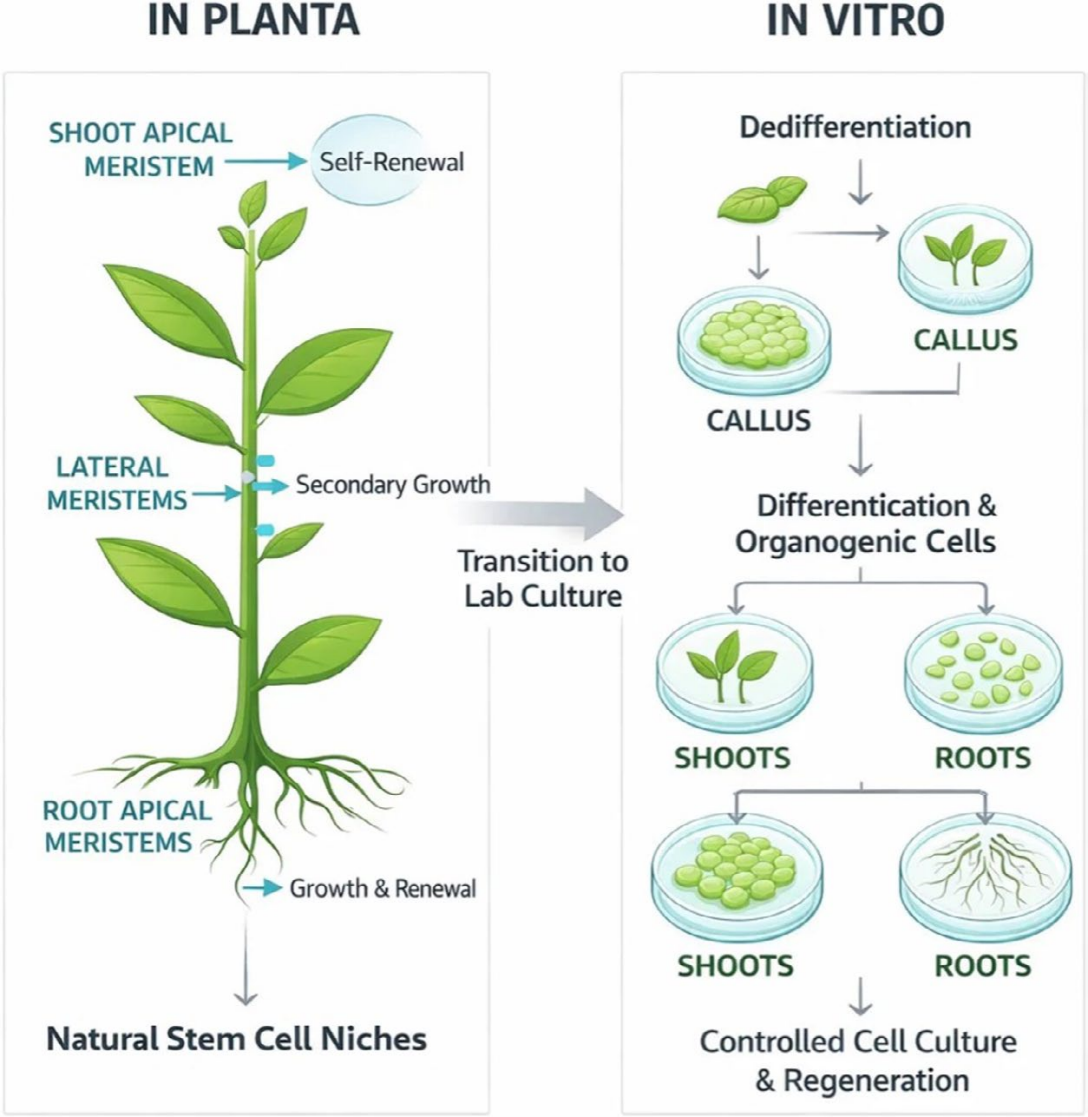
Comparison of plant stem cell processes in vivo (In Planta) and in vitro (laboratory culture conditions). The left panel illustrates the natural organization of plant stem cells within the plant, highlighting the shoot apical meristems, lateral meristems, and root apical meristems, each responsible for self‐renewal and growth. The right panel depicts the process of dedifferentiation in vitro, where plant tissues are reprogrammed into undifferentiated cells to form a callus. This callus undergoes further self‐renewal, as well as dedifferentiation and organogenesis, resulting in the production of shoots, roots, and stem cells in culture. These processes demonstrate the potential for plant stem cells to be cultivated and directed into specific developmental pathways under controlled conditions.

Another interesting example comes from an Italian research group that successfully cultured *Syringa vulgaris* plant cells under sterile laboratory conditions [1]. These cell suspensions, obtained from callus cultures, showed strong antioxidant activity and were able to inhibit lipoxygenase and 5‐alpha reductase, which are important targets for anti‐hair loss and anti‐inflammatory effects. In addition, these cells also showed strong anti‐tyrosinase activity, leading to visible skin‐whitening properties [14] (Figure 1).

New and creative uses of plant stem cells have also been reported in French research, where in vitro cultured cells of *Leontopodium alpinum* or Edelweiss were used to produce cosmetic products that help restore cell balance in aging skin, increase energy and metabolic activity, and improve skin smoothness in sagging areas [31]. Other patented studies, such as those by Ringenbach and colleagues, used undifferentiated *Marrubium vulgare* cells to prepare cosmetic formulations designed to improve skin quality, mainly by reducing visible skin flaws and tightening enlarged pores [14]. In 2016, an Italian research group developed a cosmetic active ingredient using somatic embryos from *Citrus limon* , *Lotus japonicus*, and *Rosa gardenia*. Their work focused on how these plant extracts affect genes linked to skin aging and rejuvenation, and the results showed good potential for activating antiaging pathways in skin cells [32]. In a similar way, Berry et al. patented the application of stem cell cultures from *Camellia sinensis* or the tea plant to create a product that helps prevent skin dryness and protects against UV radiation. This formulation also showed anti‐inflammatory effects and helped protect skin cells from UV‐related damage [14].

Plant stem cells have also been applied in the development of biomimetic lipids. One example is OLEA VITAE produced by Vytrus Biotech, which is obtained from wild olive tree stem cells. The phytolipid fraction in this product imitates the role of natural cellular lipids and provides anti‐wrinkle, firming, and rejuvenating effects by improving mitochondrial activity and increasing the production of important skin structural proteins [14]. The growing interest in the cosmetic use of plant stem cells reflects a broader movement toward using biotechnology to develop more effective, sustainable, and innovative skincare products. The use of these advanced technologies holds strong promise for the future of cosmetic formulations, helping to improve both skin appearance and overall skin health (Table 1).

**TABLE 1:** Benefits of plant stem cells in cosmetics.

Functions	Active compound	Primary benefit	Skin effect	Example plant	References
Antioxidant protection	Polyphenols, flavonoids	Neutralization of reactive oxygen species	Reduced oxidative stress and delayed photoaging	*Malus domestica*	[33]
Antiaging support	Phenolic acids, peptides	Protection of skin stem cells from environmental stress	Improved skin longevity and reduced wrinkle formation	*Argania spinosa*	[11, 34]
Barrier reinforcement	Phytosterols, lipids	Enhancement of epidermal barrier integrity	Improved skin hydration and resilience	*Vitis vinifera*	[35]
Anti‐inflammatory action	Triterpenes, flavonoids	Reduction of inflammation‐induced skin damage	Calmer skin and reduced erythema	*Centella asiatica*	[36]
UV‐induced stress protection	Anthocyanins	Protection against UV‐related cellular damage	Prevention of premature photoaging	*Camellia sinensis*	[37]
Environmental stress defense	Secondary metabolites	Increased resistance to pollution‐related oxidative damage	Enhanced skin defense mechanisms	*Leontopodium alpinum*	[38]

## 3 Research on Antioxidant and Antiaging Benefits of Stem Cell Extracts

Kinetin, a cytokinin abundantly produced in raspberry stem cells, is among the most effective substances for slowing the aging of human cells [14, 39]. This naturally occurring antioxidant protects proteins and nucleic acids from oxidative damage and other types of harm. Kinetin works in two major ways: it forms complexes with copper (II) ions, which activate superoxide dismutase, a crucial enzyme in the body's antioxidant defense, and it stimulates the production of enzymes that repair DNA damage and protect against oxidative stress. By preventing the formation of 8‐oxo‐dG, an oxidative marker of DNA damage, kinetin safeguards cellular DNA. Additionally, as a natural growth hormone, it improves the epidermal barrier function, promotes keratinocyte production, and reduces water loss through the skin, making it vital for enhancing skin stem cell function [40]. Cytokinins, including kinetin, regulate various plant growth processes [41], although their exact functions are difficult to define due to their interaction with other hormones and environmental factors, as well as the slow pace of their physiological effects.

Tomatoes, rich in antioxidant compounds such as lycopene, ascorbic acid, flavonoids, and phenolic acids, have also shown promise in protecting the skin from heavy metal toxicity [42]. Tomato stem cell extracts contain phytochelatins, which are strong metal‐binding proteins that prevent collagen damage induced by heavy metals by inhibiting collagenase activity [43]. Furthermore, stem cells from *Coffea bengalensis* have demonstrated the ability to encourage skin cell regeneration by stimulating fibroblasts to produce collagen [44]. These findings support the role of plant stem cells in anti‐aging by fostering fibroblast activity, promoting skin regeneration, and repairing damaged DNA.

Glycerin extracts from ginger ( *Zingiber officinale* ) leaf stem cells, developed by Naolys in France, also exhibit notable antiaging effects [45]. In a clinical trial involving 22 women, skin texture improved, pores shrank by 50%, and a matte effect was observed, with a 15% reduction in gloss and 19% in sebum production within just 6 days of use [46]. In vitro studies showed that these extracts increased elastin and fiber synthesis while reducing sebum production [47]. Naolys has also developed glycerin extracts from the stem cells of *Iris pallida* (sweet iris), *Olea europaea* (olive tree), Hibiscus rosa (Chinese hibiscus), and *Camellia sinensis* (green tea), all of which demonstrate antiaging effects on the skin [11, 14].

Extracts from *Buddleja Davidii* stem cell cultures, rich in phenylpropanoid glycosides like verbascoside, have demonstrated strong antioxidant properties [48]. Similarly, stem cell cultures from *Syringa vulgaris* (lilac) are abundant in phenylpropanoids, particularly isoverbascoside, known for their antioxidant activity [49, 50]. Studies on tomato stem cell extracts have shown that they contain higher concentrations of beneficial flavonoids and phenolic acids compared to the fruit itself, exhibiting superior overall antioxidant activity [51]. In further research, *Raspberry* stem cell extracts demonstrated potent antioxidant activity, with polyphenolic compounds such as ferulic acid and quercetin rhamnoside being the most common components [52]. Various plant stem cell extracts, including those from *Paper Mulberry*, *Grape*, *Magnolia*, *Green Tea*, and *White Ginseng*, have shown impressive antioxidant properties, with the white ginseng and green tea stem cell extracts proving particularly effective in neutralizing free radicals and scavenging DPPH radicals [53, 54].

An investigation by Vichit and Saewan utilized rice seed stem cells from varieties such as Munpu, Hommali 105, and Niawdum to assess their antiaging potential [55]. Rice contains bioactive compounds such as vitamin E, γ‐oryzanols, and anthocyanins, which contribute to health benefits like reducing atherosclerotic plaque and inhibiting cancer cell growth [56, 57]. Rice callus cultures have shown enhanced antioxidant activity and significant antiaging effects, particularly in promoting keratinocyte proliferation and reducing inflammation [55, 58]. Red rice callus extracts, when applied topically, were shown to improve skin elasticity and moisture while reducing melanin production. A clinical study with volunteers demonstrated that a 5% red rice stem cell extract product significantly improved skin hydration and flexibility [58]. Similarly, rice stem cells treated with plasma therapy showed an increase in human fibroblast proliferation and improved collagen synthesis, suggesting the potential of rice stem cells for skin regeneration [59].

Another study investigated the use of *Olea europaea* (olive tree) stem cell extracts incorporated into a cosmetic cream. This extract was found to reduce erythema and hyperpigmentation caused by laser treatments while improving skin elasticity. Biopsy samples from treated skin indicated significant regenerative activity, reinforcing the efficacy of plant stem cell‐based products in promoting skin rejuvenation and repair [60]. These studies collectively underscore the considerable potential of plant stem cell extracts in antiaging therapies, offering both antioxidant and regenerative benefits to the skin. These natural sources of bioactive compounds not only combat oxidative stress but also promote skin renewal, collagen production, and overall skin health (Table 2).

**TABLE 2:** Commercial applications of plant stem cells in skincare.

Plant extract	Product formulation	Skin care benefit	Commercial use	Research outcome	References
Apple stem cells	PhytoCellTec *Malus domestica*	Anti‐wrinkle effects	Anti‐aging creams and serums	Reduces wrinkle depth by 8%–15% after 2–4 weeks	[18, 61]
Grape stem cells	PhytoCellTec Solar Vitis	UV protection, antioxidant effects	Sun lotions and skincare	Enhances colony formation, UV protection	[14, 62, 63]
Edelweiss stem cells	LACCE (*Leontopodium alpinum*)	Skin regeneration, antiaging	Face creams and antiaging treatments	Enhances skin barrier function	[64, 65]
Citrus stem cells	Somatic embryos from *Citrus limon*	Promotes antiaging processes	Skin rejuvenation products	Promotes gene expression for skin rejuvenation	[9, 32]

## 4 Exploring Stem Cell Therapies for Wound Healing and Skin Repair

Several studies have investigated the wound healing effects of fruit extracts, especially those obtained from *Rhus coriaria* , in skin injury models. A study by Gabr and Alghadir examined an aqueous extract of *R. coriaria* fruits that contains active compounds such as gallic acid, tannins, myricetin, and quercetin. Their findings showed that topical application of this extract could speed up wound healing in both infected and non‐infected rat skin. This effect was mainly linked to its ability to control inflammation by increasing the activity of matrix myeloperoxidase and metalloproteinase 8 [14, 66]. In another in vivo study, a hydrogel prepared from an ethanolic extract of *R. coriaria* fruits was applied to rat wounds and was found to stimulate hydroxyproline and nitric oxide production, thereby accelerating healing [66, 67].

Additional research by Yu Jin Won et al. showed the beneficial effects of extracts obtained from *Rosa damascena* stem cells (RSCs) on skin health. Their study focused on exosome‐like particles (RSCEs) released from cultured RSCs and showed that these particles were non‐toxic to human dermal papilla cells. The RSCEs helped close scratch wounds and increased the proliferation of human dermal fibroblasts. They also reduced melanin production in melanocytes and lowered IL‐6 levels in Raw264.7 cells activated with lipopolysaccharide (LPS) [68, 69]. These results suggest that RSCEs have multiple useful effects, including supporting fibroblast growth, regulating pigmentation, and reducing inflammation, making them promising for aesthetic skin treatments.

In another investigation, Cho et al. studied the effects of *Leontopodium alpinum* or Edelweiss callus culture extract (LACCE) on skin regeneration. Their in vitro and in vivo experiments showed that LACCE improved skin and facial tissue regeneration by affecting molecular pathways in human keratinocytes [64, 65]. RNA sequencing analysis revealed that LACCE increased the expression of genes related to keratinization and cornification, which are important for a strong skin barrier. At the same time, it reduced stress‐related genes and increased the expression of genes involved in developmental regulation, suggesting its strong potential for use in antiaging cosmetic products [31, 65].

Guidoni et al. also examined stem cells derived from *Coffea canephora* for their anti‐inflammatory and tissue healing effects. They prepared a liposomal formulation from *C. canephora* leaf stem cells (LSCECC) and studied its wound healing ability using a rat model with full‐thickness skin wounds [70]. The wounds treated with LSCECC showed faster re‐epithelialization and significant wound size reduction of 36.4%, 42.4%, and 87.5% after 7, 10, and 14 days, respectively. These wounds also showed better granulation tissue formation, balanced inflammatory response, and improved scar quality. This healing activity was associated with increased production of growth factors, such as transforming growth factor‐beta and vascular endothelial growth factor, as well as higher collagen fiber formation and organization [31]. Overall, these studies show the growing interest in plant stem cell extracts as powerful agents for skin regeneration and wound healing due to their ability to repair tissue, control inflammation, and improve skin barrier function.

Meanwhile, the application of stem cells for wound healing and skin repair faces major regulatory and ethical challenges. The regulatory use of stem cells for wound healing and skin repair is significantly constrained by the legal definition of cosmetic products within the European Union. Under Regulation (EC) No. 1223/2009, a cosmetic product is defined as a substance or mixture intended to be placed in contact with the external parts of the human body with the exclusive or main purpose of cleaning, perfuming, changing appearance, protecting, keeping in good condition, or correcting body odors [71, 72]. Importantly, this definition explicitly excludes products intended to treat or heal disease or to restore, correct, or modify physiological functions, activities that fall within the scope of medicinal products or medical devices. As stem cell‐based therapies for wound healing and skin repair exert their effects through biological activity at the cellular and molecular levels—often involving tissue regeneration, immunomodulation, and angiogenesis—they are generally classified as advanced therapy medicinal products (ATMPs) rather than cosmetics under EU law [73]. Consequently, such products are subject to stringent regulatory pathways involving clinical trials, manufacturing under Good Manufacturing Practice (GMP), and centralized authorization procedures [74]. These regulatory boundaries limit the incorporation of viable stem cells or stem cell–derived regenerative claims in cosmetic formulations, necessitating that cosmetic applications remain confined to non‐therapeutic, surface‐level effects and substantiated claims consistent with the cosmetic regulatory framework [75, 76].

## 5 Plant Stem Cells and Their Role in UV Protection

Secondary metabolites produced from biotechnologically grown meristem cells have shown a strong ability to protect human skin from UV damage in both in vivo and in vitro studies, especially in keratinocyte cultures. These metabolites have special molecular structures made of condensed aromatic 5 and 6 carbon rings with several hydroxyl groups, which makes them effective photoprotective agents. This structure allows them to absorb UV‐A and UV‐B radiation efficiently and act similarly to sunscreens without causing harmful photochemical reactions. Many glycosylated metabolites also show higher photo‐stability and resistance to UV‐A degradation [77].

Active compounds derived from meristem plant cells have also proven effective in protecting skin fibroblasts and endothelial cells against UV and bacteria‐related damage. When keratinocytes were exposed to bacterial lipopolysaccharides or inflammatory cytokines, treatment with these active compounds significantly reduced reactive oxygen species and inflammatory cytokine production. These effects were similar to the action of non‐steroidal anti‐inflammatory drugs and topical corticosteroids [78, 79]. Plant cell cultures treated with suitable elicitors are also a rich source of low‐molecular‐weight antioxidants that help regulate oxidative stress directly and indirectly. Because these small molecules are compatible with human skin cells, they are well‐suited for regulating oxidative balance in the skin [80]. For example, *Vitis vinifera* (grapes) contains high levels of phenolic acids, catechins, and anthocyanins, which act as strong free radical scavengers and antioxidants [81].

**FIGURE 2:**
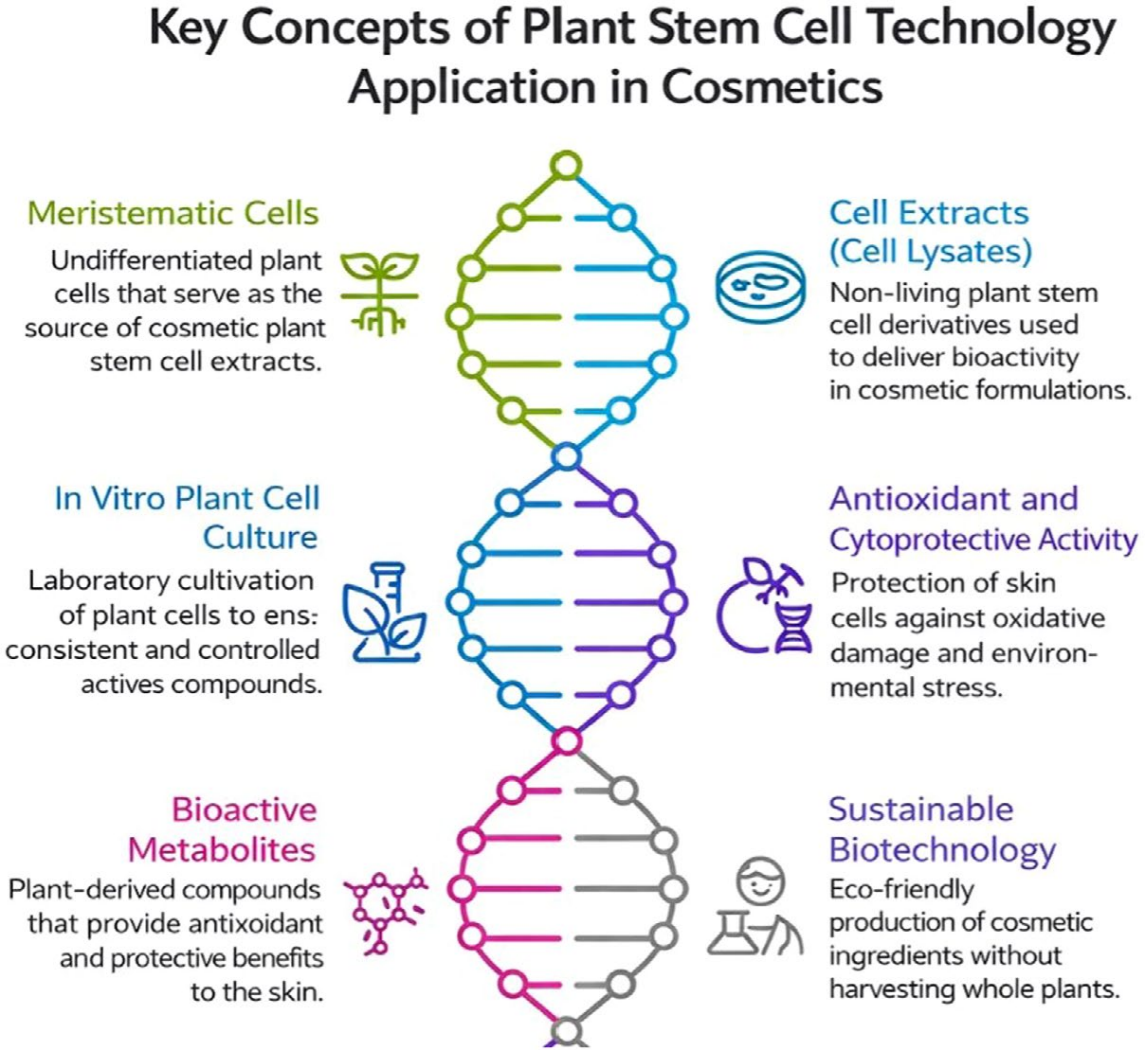
Schematic overview of key concepts in plant stem cell technology for cosmetic applications, depicting meristematic cells as the initial source of undifferentiated plant material, followed by in vitro plant cell culture for the controlled and reproducible production of active compounds. The generation of bioactive metabolites and their incorporation into cosmetic formulations as non‐living cell extracts (cell lysates) contribute to antioxidant and cytoprotective activity by helping protect skin cells from oxidative and environmental stress.

Studies also showed that applying a grape stem cell extract known as PhytoCellTec Solar Vitis to epidermal stem cells increased their colony formation efficiency by 86% [62]. In addition, this extract protected the cells from UV damage, which normally caused about a 50% reduction in colony formation in untreated cells. In a clinical study, a sunscreen lotion containing grape stem cell extract was able to reduce UV‐induced erythema on the skin [14, 63] (Figure 2).

## 6 Aging of Hair Follicles

Apple stem cells, especially those derived from the *Uttwiler Spätlauber* apple variety, have been widely studied for their ability to slow skin aging and improve hair follicle health. In a study conducted by Mibelle AG Biochemistry, the effects of apple stem cell extract on hair follicles during the anagen growth phase were tested. In this experiment, one group of hair follicles received only growth nutrients, while another group was treated with 0.2% apple stem cell extract. To create proper growth conditions, both groups were protected using skin fragments obtained after a facelift procedure. After 14 days, the hair follicles treated with apple stem cell extract continued growing, while the untreated follicles began to shrink due to aging and apoptosis. These findings clearly show that plant stem cell extracts can support prolonged hair growth and slow down follicle aging, making them a promising option for hair rejuvenation therapies.

## 7 Challenges in Ensuring the Safe and Effective Use of Stem Cells in Cosmetics

Overall, the use of stem cells in cosmetic products is limited by scientific, technical, and regulatory constraints. From a scientific perspective, viable stem cells are inherently unstable outside controlled laboratory conditions and cannot survive standard cosmetic formulation processes, which involve preservatives, emulsifiers, and long‐term storage. Moreover, intact stem cells cannot penetrate the skin barrier, and claims of regeneration or tissue repair would imply biological activity beyond the superficial effects permitted for cosmetics.

Using clear and correct terminology is very important when making claims about stem cell cosmetic products. In most cases, when companies use the term plant stem cell, they actually refer to extracts taken from primitive plant cells rather than live stem cells themselves. Even though advertisements may suggest otherwise, most skincare products only contain stem cell extracts and not actual living stem cells. In addition, cultured plant stem cells cannot be directly added into cosmetic formulas, since they cannot survive cosmetic processing or preservatives, cannot penetrate the skin due to their size and rigid cell walls, and would not remain biologically active or functional in human skin [14].

The direct marketing of stem cell‐based products to consumers is now widespread, with many companies worldwide offering them. Some of these products actually contain stem‐related materials but are not always properly regulated or clinically tested. Others are labeled as stem cell products but do not actually contain any stem cells. Since stem cells are biological materials, they must be handled with great care [75, 82]. Labs that prepare stem cell‐related cosmetic ingredients must follow strict regulations. Clear production steps must also be defined, such as tissue source, collection procedures, cell isolation, culture methods, phenotypic identification, and application methods. Also, detailed clinical studies are required to prove the safety of these products [75, 83]. These limitations are compounded by the regulatory framework governing cosmetics, which does not require mandatory clinical trials prior to market entry. Unlike medicinal products, cosmetic products are assessed primarily for safety, not therapeutic efficacy [84, 85]. While manufacturers must compile a Cosmetic Product Safety Report and ensure that claims are truthful and substantiated, this substantiation does not necessarily involve randomized controlled clinical trials and may rely on in vitro data, ingredient‐level evidence, or consumer perception studies [75, 86]. As a result, stem cell‐related claims in cosmetics are typically framed around indirect or protective effects—such as antioxidant activity or support of skin appearance—rather than true regenerative outcomes. To protect the public, stem cell‐based cosmetic products must strictly comply with advertising rules and safety guidelines set by national drug and medical authorities. This issue is not limited to stem cell cosmetics only, but applies to the whole cosmetic industry. Many new products enter the market before they undergo sufficient clinical testing, mainly because regulatory systems cannot always keep pace with rapid industry growth. To follow market demands and social media trends, some companies may release products that are not fully approved or tested. Therefore, educating consumers about approval processes, safety standards, and possible risks is very important so they can make better choices and report any suspicious practices [87].

## 8 Conclusion

Stem cell‐related technologies have expanded the scope of cosmetic applications by introducing bioactive extracts and cell‐derived factors that support skin protection, antiaging, and improved skin appearance, and by introducing innovative concepts into cosmetic science, particularly through the use of stem cell‐derived extracts and bioactive compounds that support skin appearance and protection. While true regenerative effects remain outside the cosmetic domain, continued progress in biotechnology, ingredient processing, and mechanistic understanding has enabled more sophisticated and scientifically grounded cosmetic formulations. These developments underscore the growing role of stem cell–inspired innovations in cosmetics, provided they remain aligned with regulatory definitions, safety requirements, and evidence‐based claims. However, their application is fundamentally limited by biological feasibility and strict regulatory boundaries that distinguish cosmetic products from medicinal therapies. Current cosmetic formulations cannot incorporate viable stem cells nor legitimately claim regenerative or wound‐healing effects without exceeding the legal definition of a cosmetic product, especially under the EU regulatory framework. Moreover, the absence of mandatory clinical trials for cosmetics places greater responsibility on manufacturers to ensure that claims are scientifically substantiated and clearly communicated. Future progress in this field will depend on maintaining a clear distinction between cosmetic and therapeutic intent and on improving transparency.

## Author Contributions

**Farid KarkonShayan, Nasim Saderi, Iman Morshedi, Behnaz Ghamari, and Sepideh KarkonShayan:** conceptualization, writing – original draft, writing – review and editing, and supervision.

## Funding

The authors have nothing to report.

## Ethics Statement

The authors have nothing to report.

## Consent

The authors have nothing to report.

## Conflicts of Interest

The authors declare no conflicts of interest.

## Data Availability

Data sharing is not applicable to this article as no datasets were generated or analyzed during the current study.
